# Combined Effect of Dietary Acid Load and Cardiometabolic Syndrome on Bone Resorption Marker among Post-Menopausal Women in Malaysia

**DOI:** 10.21315/mjms2024.31.2.10

**Published:** 2024-04-23

**Authors:** Sook Yee Lim, Yoke Mun Chan, Yit Siew Chin, Mohd Shariff Zalilah, Vasudevan Ramachandran, Manohar Arumugam

**Affiliations:** 1 Department of Dietetics, Faculty of Medicine and Health Sciences, Universiti Putra Malaysia, Selangor, Malaysia; 2 Faculty of Applied Sciences, UCSI University, Kuala Lumpur, Malaysia; 3 Research Center of Excellence Nutrition and Non-communicable Diseases, Faculty of Medicine and Health Sciences, Universiti Putra Malaysia, Selangor, Malaysia; 4 Malaysian Research Institute on Ageing, Universiti Putra Malaysia, Selangor, Malaysia; 5 Department of Nutrition, Faculty of Medicine and Health Sciences, Universiti Putra Malaysia, Selangor, Malaysia; 6 Department of Medical Science, Faculty of Health Sciences, University College MAIWP International, Kuala Lumpur, Malaysia; 7 Department of Conservative Dentistry and Endodontics, Saveetha Dental College and Hospitals, Saveetha Institute of Medical and Technical Sciences, Saveetha University, Chennai, India; 8 Department of Orthopedics, Faculty of Medicine and Health Sciences, Universiti Putra Malaysia, Selangor, Malaysia

**Keywords:** dietary acid load, interaction effect, cardiometabolic syndrome, vitamin D, CTX1

## Abstract

**Background:**

This study aimed to investigate factors associated with bone resorption status and determine the independent and interactive effects of dietary acid load (DAL) and cardiometabolic syndrome (CMS) on bone resorption in post-menopausal women.

**Methods:**

Overall, 211 community-dwelling post-menopausal women were recruited from the National Council of Senior Citizens Organization, Malaysia. DAL was estimated using the potential renal acid load from the food frequency questionnaire. Sleep quality was assessed using the Pittsburgh Sleep Quality Index (PSQI) and smoking behaviour was assessed using the Global Adult Tobacco Survey 2011. Serum 25(OH) vitamin D levels were determined using the ADVIA Centaur vitamin D assay and serum C-terminal telopeptides of type I collagen (CTX1) were used as surrogate markers to assess bone resorption. CMS was determined based on the harmonised criteria.

**Results:**

Age (*β* = −0.145, *t* = −2.002, *P* < 0.05) was negatively associated while DAL (*β* = 0.142, *t* = 2.096, *P* < 0.05) and sleep quality (*β* = 0.147, *t* = 2.162, *P* < 0.05) were positively associated with CTX1. Height was positively correlated with CTX1 (*r* = 0.136, *P* <0.05). Conversely, other variables (CMS traits, CMS, serum 25(OH) vitamin D level, years of menopause, years of education and physical activity) were not significantly associated with CTX1 levels. There was no significant interaction between DAL and CMS on bone resorption.

**Conclusion:**

Our findings propose that high DAL, but not CMS, is a potential risk factor for bone resorption. The analysis did not demonstrate the combined effects of DAL and CMS on bone resorption.

## Introduction

Bones are constantly being remodelled; first broken down (resorption) and then rebuilt (formation) in tight co-ordination to maintain skeletal integrity. Bone loss uncoupled from bone formation results in reduced bone mass, as in the case of post-menopausal women, making them more prone to developing osteoporosis (1, 2). Osteoporosis is an age-related chronic disease characterised by low bone mass and deterioration of bone quality affecting > 200 million people worldwide (3). A sharp decline in oestrogen levels during the post-menopausal period may reduce bone formation and increase bone turnover (4), leading to a higher risk of hip fractures.

Ample evidence has shown that degenerative aging processes are the major cause of cardiometabolic syndrome (CMS) (5), a constellation of metabolic factors that increases the risk of cardiovascular disease and type 2 diabetes. CMS is prevalent among post-menopausal women, with a prevalence of 22.2% (6) and 29.5% (7) in Brazil, 46% in India (8), 57.9% in Western Algeria (9) and 61.7% in Spain (10). The prevalence of CMS is relatively high in post-menopausal women regardless of the population (11). CMS and osteoporosis are multifactorial diseases resulting from interactions between environmental and genetic factors. Environmental factors such as low body mass index (BMI), smoking, a sedentary lifestyle, alcohol and caffeine intake, inadequate calcium and vitamin D intake, steroids, thyroid hormones, frusemide medication for chronic diseases, serum ferritin levels, and endocrine diseases may be associated with osteoporosis risk (12, 13). The detrimental effects of CMS on bones may be attributed to the shared effects of pro-inflammatory, pro-oxidative and pro-calciuric body environments (14).

The acid-base balance hypothesis proposes that vegetables and fruits are rich in basic elements like calcium, phosphate, potassium and magnesium, whereas animal sources may increase proton (H+) ions. Habitual dietary intake of acidic foods may lead to an increased net acid load in the body. Under long-term exposure to an acidic environment, bones may act as buffer systems to release calcium salts and maintain the acid-base balance in the body, which may reduce bone mineral density (BMD) (15). Studies have shown that an acidic diet rich in animal sources and low in vegetables and fruits can lead to chronic metabolic acidosis, causing calcium loss from bones (16–18). Current evidence shows that excessive dietary acid load (DAL) is a risk factor for osteoporosis and CMS through excess acid supply (16, 19, 20).

Although the acid-base balance theory hypothesises that habitual high DAL levels are associated with a higher risk of osteoporosis, evidence on their influence on bone health has been inconsistent. These contradictory findings may be due to a lack of consideration of the links between DAL and diabetes (18), hypertension (21), obesity (21), cardiovascular disease (22), cortisol levels (23) and overall well-being in bone health. The mechanisms underlying these associations have not been completely elucidated. Conversely, several studies have investigated the association of osteoporosis with DAL (15, 24–28) and BMD with CMS (29–31) but research on the combined effect of DAL and CMS on osteoporosis development is scarce. Thus, our study aimed to investigate the factors associated with bone resorption status and determine the relationship between DAL and CMS on bone resorption among post-menopausal women.

## Methods

### Study Design and Respondents' Recruitment

This analytical cross-sectional study included 211 healthy community-dwelling post-menopausal Chinese women. The sample size was calculated using the G*Power software (www.gpower.hhu.de). The effect size was 0.15, considered to be ‘medium’ using Cohen’s (32) criteria. With a significant criterion of α = 0.05 and power = 0.95, the minimum sample size needed with this effect size is 208 for linear multiple regression. Thus, the obtained sample size of 211 was adequate to test the study hypotheses. The methodology of this study has been previously published elsewhere (33–35). Written informed consent was obtained from all respondents prior to enrolment in the study.

### Research Instrumentation

Instruments used for data collection included questionnaires, physical examination and biochemical measurement.

#### Questionnaires

A pre-tested structured questionnaire was used to obtain information on socio-demographics, medical history, lifestyle, physical activity level and dietary intake of the respondents. The smoking behaviour of respondents was assessed using the Global Adult Tobacco Survey 2011 (GATS 2011) (36). GATS has been adopted and implemented in a nationwide cross-sectional survey in Malaysia (37) and has proven able to provide reliable data on various dimensions of tobacco control, such as exposure to second-hand smoke and willingness to quit smoking. Assessment on physical activity of the respondents was adapted from the World Health Organization (WHO) Global Physical Activity Questionnaire version 2.0 instrument (GPAQv2) (38), while the sleep quality of the respondents during the past month was assessed using the Pittsburgh Sleep Quality Index (PSQI) (39). A global PSQI score > 5 yielded a diagnostic sensitivity of 89.6% and specificity of 86.5% (kappa = 0.75, *P* < 0.001) in examining good and poor sleepers (39).

Habitual food intake was assessed using a validated 165-item semi-quantitative food frequency questionnaire (FFQ) (40) adapted from the Malaysian Adult Nutrition Survey (MANS) (41). The food items were commonly consumed by Malaysians and were categorised into 14 food groups. Respondents were requested to report the number of servings consumed each time, according to the suggested standard serving size (42). The portion sizes consumed by the respondents were converted to grams based on published household measurements (42). Furthermore, the respondents were asked to report the frequency of intake of each food item on a daily, weekly or monthly basis. Nutrient and energy intakes were calculated using Nutritionist Pro™ Diet Analysis (Axxya Systems, Stafford, TX, USA) software, with the Nutrient Composition of Malaysian Foods (43) and Singapore Food Composition Database (44) as the primary dietary databases.

The respondents’ DAL was calculated using the potential renal acid load (PRAL) (45), which is an equation based on the ionic balance of the nutrients and intestinal absorption rates of protein and four main minerals (phosphorus, potassium, magnesium and calcium), as well as sulphate production from protein metabolism (45). A positive PRAL score indicates acid load or acid-forming potential, while negative scores reflect alkaline load or alkaline-forming potential (45). To date, PRAL calculation is the established method for estimating net acid excretion and has been reported to confidently predict urinary net acid excretion despite reporting bias (46).

#### Physical Measurements

Physical characteristics including anthropometric measurements (weight, height and waist circumference [WC]) and blood pressure (BP) were assessed by trained researchers. Measurements were taken immediately after the respondents completed the registration and before administering the questionnaire.

Height, weight and WC were measured using a portable stadiometer (SECA, Hamburg, Germany), a digital weighing scale (TANITA Technology, Tokyo, Japan) and Lufkin tape (Apex Tools, Sparks, MA, USA), respectively. All measurements were performed using standardised techniques (47, 48). The WC classification was based on the WHO/International Association for the Study of Obesity (IASO)/International Obesity Task Force (IOTF) criteria (49). Respondents’ BP was measured using a digital automatic BP monitor (OMRON HEM-907; Omron Healthcare, Kyoto, Japan).

#### Biochemical Measurements

Fasting blood samples (10 mL) were drawn from the antecubital veins of the participants into ethylenediaminetetraacetic acid (EDTA) (Becton Dickinson, NJ, USA) and plain tubes by certified phlebotomists. The tubes were immediately sent to a laboratory for blood glucose, lipid profile, vitamin D and CTX1 analyses. Fasting plasma glucose levels were determined by the hexokinase method using an Olympus AU analyser (Beckman-Coulter, Inc., Fullerton, CA, USA), while lipid profile was determined using commercially available kits on a Hitachi 704 Analyser (Roche Diagnostics, Tokyo, Japan). Total cholesterol and triglyceride levels were analysed using the cholesterol oxidase/peroxidase and glycerol phosphate oxidase/peroxidase methods, respectively. Serum CTX1 was assessed by a fully automated analyser (Elecsys 2010, Roche Diagnostics, GmbH, Mannheim, Germany) while serum 25(OH) vitamin D levels were determined by using the Siemens ADVIA Centaur Vitamin D Total assay (Siemens, Tarrytown, NY, USA), with the analytical measuring range 4.2 ng/mL– 150 ng/mL (10.5 nmol/L–375 nmol/L). Although there is no consensus on the definition of vitamin D deficiency, serum 25(OH) vitamin D levels < 50 nmol/L are widely used in studies and recommended by the US Institute of Medicine (50). To date, there is no international consensus on the diagnostic criteria or cut-off points for bone resorption markers. On the other hand, the universal harmonised criteria were adopted to confirm the CMS diagnoses of respondents in lieu of being the most suitable for Asian populations (51–53). An individual is defined as having CMS in the presence of at least three risk factors like abdominal obesity, hypertension, hyperglycaemia and dyslipidaemia (51).

### Statistical Analyses

Statistical analyses were performed using IBM SPSS 22.0 (IBM Corp., Armonk, NY, USA), with the level of significance set at *P* < 0.05. Data quality was assessed using SPSS to remove outliers and test for normality. Continuous variables such as age, years of menopause, years of education, physical activity level, weight, height, waist circumference, PRAL, serum 25(OH) vitamin D levels and CTX1 were expressed as mean ± standard deviation, while categorical variables were expressed as frequencies and percentages. Bivariate analyses of Pearson’s correlations were used to determine the relationships between the rate of bone resorption and DAL, anthropometric parameters (waist circumference, BMI, weight and height), blood pressure (systolic and diastolic blood pressure), biochemical indices (fasting blood glucose, HDL-C, triglycerides and serum vitamin D), socio-demographic background (age and years of education), years of menopause and lifestyle factors (sleep quality and physical activity level).

A three-step hierarchical multiple linear regression (MLR) was used to determine the relative importance of a set of variables comprising diet and diseases (PRAL and CMS), as well as the interaction term (PRAL × CMS), with the respondent’s risk factors (age, years of education, height, PSQI and serum 25(OH) vitamin D levels) in predicting bone resorption. Before the analysis, several assumptions such as linearity, homoscedasticity, independence of error terms, normality of the error distribution and absence of multi-collinearity were met based on the steps provided by Ho (54).

## Results

### Characteristics of the Respondents

Table 1 shows the respondents’ socio-economic, demographic and anthropometric characteristics. The mean age of the respondents was 66.7 ± 6.6 years old, with three quarters of them in the ‘young-old’ category. The mean duration of menopause was slightly longer than 15 years and > 75% of the participants were married. The mean duration of education was < 10 years. While approximately 40% of the respondents were overweight and obese according to BMI, half had central obesity with excessive WC (Table 3). The respondents’ lifestyle variables are listed in Table 2. Approximately 99% of respondents were non-smokers. In general, the respondents were active and more than 60% met the recommendations for physical activity. Nevertheless, sedentarism was prevalent, with 30.8% engaging in sedentary activities for more than 4 h per day. More than half of the respondents were good sleepers.

The estimated DAL, vitamin D status, CMS and rate of bone resorption of the respondents are presented in Table 3. Means PRAL was 13.8 ± 19.08 mEq/day. A deficiency in vitamin D was evident, with 82% of respondents exhibiting either serum 25(OH) vitamin D deficiency or inadequate levels. Approximately half of the respondents had CMS. Elevation of systolic blood pressure and hyperglycaemia were the most dominant components of CMS among the respondents, seen in approximately 70% and 60% of them, respectively. A quarter of the respondents had hypertriglyceridaemia. Being the surrogate marker of the rate of bone loss, mean CTX1 was 0.5 ± 0.20 μg/L (range: 0.05 μg/L–0.90 μg/L).

### Correlations between Variables and Bone Resorption

As shown in Table 4, CTX1 was weakly but significantly correlated with age (*r* = −0.180, *P* = 0.009) and height (*r* = 0.136, *P* = 0.049), with younger and taller participants having a higher rate of bone resorption. Conversely, there were no significant correlations between CTX1 and DAL (PRAL), CMS traits (WC, SBP, DBP, FBG, HDL-C and triglyceride), serum 25(OH) vitamin D levels, socio-demographic background (years of education), sleep quality, physical activity level or weight.

### Independent and Combined Effect of DAL and CMS on Bone Resorption

Table 5 shows that the set of adjusted variables accounted for 7.3% of the variance in the rate of bone resorption. Age was negatively associated while sleep quality was positively associated with CTX1. The younger the respondents, the higher the rate of bone resorption (*β* = −0.145, *t* = −2.002, *P* = 0.047). Additionally, the poorer the sleep quality, the higher was the rate of bone resorption (*β* = 0.147, *t* = 2.162, *P* = 0.032). Conversely, years of education, height and serum 25(OH) vitamin D levels were not significantly associated with CTX1. The results revealed a significant model (*F* (6,204) = 3.207, *P* = 0.008, *R*^2^ = 0.073). In step 2 of the hierarchical multiple linear regression to evaluate the main effects of PRAL and CMS, PRAL was significantly associated with CTX1 (*β* = 0.142, *t* = 2.096, *P* = 0.037). In step 3, there was no significant interaction effect between PRAL and CMS.

## Discussion

Our findings showed that bone resorption is associated with age, height and sleep quality. In this study, younger age was associated with a higher rate of bone resorption. Increased age is an established determinant of lower BMD, which further increases the risk of fractures (3). Nevertheless, the association was less consistent when rate of bone loss was used as the dependent variable (55, 56). Several factors can cause variations in bone resorption. For instance, variations in the reduction in glomerular filtration rate (GFR) with age may affect bone resorption results. Moreover, the timing of blood sample collection is of the utmost importance in causing variation. Bone markers are affected by exercise and their concentrations can be significantly increased for 24 h–72 h after strenuous exercise (57). It is of utmost importance to note that the negative association between age and bone resorption among the respondents may be due to age-related physiological changes in BMD. Oestrogen deficiency before and at the beginning of menopause induces rapid bone resorption, and a consistent gap between bone resorption and formation results in bone loss (58). The rapid rate of bone resorption usually starts a year prior to menopause (peri-menopause) and remains high for another 3 years after menopause before a decelerating process. The rate of bone resorption for 4 years–8 years after menopause remains high which explains the rapid bone loss and greatest decline in BMD among early post-menopausal women (59).

Our findings are comparable with those of other studies that indicated that tall stature is associated with a higher risk of osteoporosis and fractures (60). In a cohort study consisting of over 92,000 American white women, women with a height of ≥ 173 cm or taller sustained hip fractures more (61); similar observations were reported in men (60). It is generally recognised that the risk of osteoporosis in adults is influenced by peak bone mass achieved during childhood and adolescence (62). Every standard deviation increase in height of the distal tibia, distal fibula and distal radius has been shown to result in a lower volumetric BMD (63, 64). Individuals with tall stature may develop wider bones with more porous and thinner cortices to maintain the strength and lightness of long bones (64). The increased rate of bone turnover after menopause may explain why taller women tend to have higher risks of osteoporosis and bone fractures.

Our findings give further credence that poor sleep quality was associated with higher rate of bone resorption (65, 66). Evolving data indicate that endocrine dysfunction and the elevation of pro-inflammatory cytokines which are related to sleep quality, are the underlying mechanisms which may cause bone deterioration (65, 67). Considering the concomitantly high prevalence of poor sleep quality and bone health among post-menopausal women, appropriate interventional studies are warranted.

Oestrogen has been shown to have protective effects against increases in blood pressure (68). A rat study showed an increase in blood pressure in ovariectomised rats but not in rats treated with oestrogen replacement therapy (69). These findings support the role of oestrogen in post-menopausal hypertension. Conversely, previous studies have reported that oestrogen has protective effects on insulin resistance and glucose homeostasis (70) and reduces lipid deposits in the endothelium with increased high density lipoprotein cholesterol (HDL-C) and reduced low density lipoprotein cholesterol (LDL-C) and cholesterol levels in the plasma (71). A decrease in oestrogen production after menopause may make women more vulnerable to metabolic diseases such as hypertension, hyperlipidaemia and diabetes, leading to an increased risk of cardiovascular disease.

In this study, the mean serum CTX1 levels of the respondents was comparable to those reported in previous studies (72, 73). Although a few studies have been conducted on CTX1 in post-menopausal women, a direct comparison cannot be made because different ethnicities may have influenced the variability of the results. For instance, Chinese women have been reported to have suboptimal bone health or higher hip fractures than Malay and Indian women in Malaysia (74, 75) and Singapore (76). Moreover, different units of CTX1 were used in different studies, making comparisons impossible. CTX1 is involved in the enzymatic degradation of type 1 collagen. It generates fragments of different molecular sizes and immunoassays detect any fragments that contained eight amino acid sequences used to raise the antibodies, making the determination of the specific molecular weight involved challenging (77).

The relationship among bone health, DAL and CMS has been extensively investigated in recent years, with inconsistent conclusions. Our findings showed that a high-acidity diet, as determined by PRAL, accentuated the rate of bone resorption, contradicting the results of previous studies. Several cross-sectional and randomised controlled trials (27, 28, 78) have shown that PRAL is negatively associated with BMD, bone quality and muscle mass, whereas others have failed to demonstrate similar association (15, 24–26, 79). These inconsistent findings may be explained by variations in genetic makeup. Despite inconsistent findings from previous research, this study supports the hypothesis that a habitually high intake of DAL may stimulate osteoclast activity and affect bone health. Previous studies have suggested several mechanisms underlying the influence of diet-induced acidosis on bone turnover (80–82). Frick and Bushinsky (80) suggested that a slight decrease in metabolic pH caused by high DAL results in the depletion of calcium from the bone by decreasing urinary calcium excretion without increasing calcium absorption in the intestine. Excess calcium excreted in urine associated with an acidic diet over time can be as high as 24 g per year or 480 g over 20 years (83). The estimated calcium loss in urine is almost half of the adult skeletal mass of calcium (83). Long-term acidity of the bloodstream and bones act as homeostatic organs to neutralise the metabolic pH, which may subsequently lead to osteoporosis.

The current study found that CTX1 was not significantly associated with the CMS criteria. To the best of our knowledge, no study has examined the association between CMS and bone resorption; hence, the discussion of the above association is based on the available evidence on CMS and BMD. Earlier studies showed negative association between CMS and BMD in post-menopausal women (30, 84). One possible explanation is that CMS and BMD reductions may share similar risk factors (85). Wong et al. (84) suggested that these two disorders share several underlying mechanisms such as calcium homeostasis regulation, receptor activator of NF-κB ligand (RANKL)/receptor activator of NF-κB (RANK)/osteoprotegerin (OPG) and Wnt-β catenin signalling. However, other studies have not reported similar findings (29, 31). These discrepancies could be attributed to genetic factors or demographic characteristics of the study respondents.

The analysis did not reveal any significant interactive effects of DAL and CMS on bone resorption. There is no clear explanation for this; however, it may be related to other risk factors which have higher contribution to the development of bone resorption. Although the present study failed to show the interaction effect between DAL and CMS on the rate of bone resorption, it provided evidence that a high-acidity diet may accentuate the risk of osteoporosis, whereas CMS fails to show an attribution to the rate of bone resorption. These findings have significant public health implications. It provides critical information for relevant authorities in the planning, implementation, monitoring and evaluation of appropriate programmes to prevent or delay the onset of osteoporosis among post-menopausal women.

This study had several limitations. First, this was a cross-sectional study; thus, we were unable to determine the causality between the consumption of a high DAL diet or CMS and the risk of osteoporosis. Second, DAL was estimated based on dietary intake. Reporting bias in the determination of food intake may also be present. Additionally, the absorption of individual ions from food varies. It is highly dependent on an individual’s gastrointestinal absorption of nutrients from food and kidney function to filter net acid excretion. Although stool specimens have been proposed as a direct measure of acid and alkali content, stool specimen collection is challenging with regard to respondents’ perspectives, concerns about hygiene and contamination, and other barriers. Thus, estimating DAL from food is still an established method for estimating net acid excretion, despite its limitations. Third, the present study included self-reported questionnaires, which may have resulted in under- or over-reporting, especially with regard to dietary intake, physical activity and sleep quality. Although verbal instruction was provided in administering the questionnaires and face-to-face interviews were performed with those who had difficulties in filling out the form, this limitation should not be underestimated. The use of only one bone resorption marker is another limitation of this study. Lastly, although potential confounders were included in the analysis, the presence of other potential confounding factors, such as gene-gene interactions or gene-diet interactions, could not be adjusted for.

## Conclusion

In conclusion, a high DAL but not CMS, is a potential risk factor for bone resorption. Our analysis did not demonstrate the combined effects of DAL and CMS on bone resorption. However, the current study extends the earlier understanding of the potential combined effects of DAL and CMS on the bone resorption rate. Taken together, these results provide new insights for scientific research to focus on other risk factors that interact with DAL in bone resorption to prevent or delay the onset of osteoporosis among post-menopausal women. For future studies, a case-control design would be helpful for identify possible predictors of bone loss and studying osteoporosis among post-menopausal women.

## Figures and Tables

**Table 1:** Socioeconomic, demographic and anthropometric characteristics of respondents (*n* = 211)

Variable	*n* (%)	Mean ± SD
Age (years old)		66.7 ± 6.6
Older adult (< 60)	27 (12.8)	
Young-old (60–74)	158 (74.9)	
Old-old (75–84)	26 (12.3)	
Years of menopause (years)		16.1 ± 7.8
5–10 years	64 (30.3)	
>10 years	147 (69.7)	
Marital status		
Single	21 (10.0)	
Married	162 (76.8)	
Divorced	6 (2.8)	
Widowed	22 (10.4)	
Education (years)		8.0 ± 4.6
Education level		
No formal education	27 (12.8)	
Primary school	68 (32.2)	
Lower secondary school	30 (14.2)	
Upper secondary school	57 (27.0)	
Tertiary: Diploma/Degree/Master/PhD	29 (13.8)	
Monthly household income*		
≤ RM2,300	95 (45.0)	
RM2,300–RM5,599	76 (36.0)	
≥ RM5,600	40 (19.0)	
Occupation		
Housewife	79 (37.4)	
Retiree	104 (49.3)	
Others	28 (13.3)	
Body weight (kg)	57.9 (9.50)	
Height (cm)	154.0 (5.44)	
BMI (kg/m^2^)	24.4 (4.02)	
Underweight (< 18.5)	10 (4.7)	
Normal (18.5–24.9)	119 (56.4)	
Overweight (25.0–29.9)	67 (31.8)	
Obese I (30.0–34.9)	9 (4.3)	
Obese II (35.0–39.9)	5 (2.4)	
Obese III (≥ 40.0)	1 (0.5)	

Note: 1 USD = RM4.29 at time of data collection (June 2017)

**Table 2:** Lifestyle characteristics of respondents (*n* = 211)

Variables	*n* (%)	Mean ± SD
Smoking		
Non-smoker	208 (98.6)	
Current smoker	2 (0.9)	
Ex-smoker	1 (0.5)	
Second-hand smoker	47 (22.3)	
Physical activities		
Total physical activity (MET-min/week)		989.7 ± 0.49
Below recommendation (< 600 MET-min/week)	79 (37.4)	
Meeting recommendation (≥ 600 MET-min/week)	132 (62.6)	
Sedentary behaviour (in minutes)		227.3 ± 140.67
< 4 h	146 (69.2)	
≥ 4 h	65 (30.8)	
Sleep quality parameters		
PSQI global score		5.7 ± 3.59
Sleep quality		
Good sleeper (≤ 5)	115 (54.5)	
Poor sleeper (> 5)	96 (45.5)	

Note: Data were presented as *n* (%) or mean ± SD

**Table 3:** Distribution of respondents according to DAL estimation, vitamin D status, CMS and CTX1

Variable	*n* (%)	Mean ± SD	Median	Range
PRAL (mEq/day)		13.8 ± 19.08		−49.49–85.32
Serum 25(OH) vitamin D (nmol/L)		37.8 ± 14.41		13.60–83.00
Deficient (< 30 nmol/L)	67 (31.8)			
Inadequate (30–50 nmol/L)	106 (50.2)			
Adequate (> 50 nmol/L)	38 (18)			
CMS traits				
WC (cm)		80.4 ± 9.25		58.40–116.70
Normal (< 80 cm)	107 (50.7)			
Abdominal obesity (≥ 80 cm)	104 (49.3)			
SBP (mmHg)		140.0 ± 20.19		92.00–214.00
Normal (< 130 mmHg)	67 (31.8)			
Elevated (≥ 130 mmHg)	144 (68.2)			
DBP (mmHg)		77.0 ± 10.22		51.00–103.00
Normal (< 85 mmHg)	162 (76.8)			
Elevated (≥ 85 mmHg)	49 (23.2)			
Fasting blood glucose (mmol/L)		6.0 ± 1.17		4.40–12.60
Normal (< 5.6 mmol/L)	86 (40.8)			
Elevated (≥ 5.6 mmol/L)	125 (59.2)			
HDL-C (mg/dL)		1.6 ± 0.36		0.80–3.00
Normal (≥ 1.3 mmol/L)	188 (89.1)			
Low (< 1.3 mmol/L)	23 (10.9)			
TG (mg/dL)		1.4 ± 0.61		0.40–3.76
Normal (< 1.7 mmol/L)	162 (76.8)			
Elevated (≥ 1.7 mmol/L)	49 (23.2)			
Presence of CMS	102 (48.3)			
Normal	109 (51.7)			
Bone resorption rate: CTX1 (μg/L)		0.5 ± 0.20		0.05–0.90

**Table 4:** Bivariate correlations between study variables and rate of bone resorption (*n* = 211)

	Rate of bone resorption marker (CTX1)	
	*r*	*P*
PRAL	0.099	0.15
WC	−0.106	0.13
BMI	−0.093	0.18
Systolic BP	−0.099	0.15
Diastolic BP	0.021	0.77
fasting blood glucose	−0.131	0.057
HDL-C	−0.085	0.22
Triglyceride	−0.023	0.74
Serum 25(OH) vitamin D	−0.090	0.20
Age	−0.180**	0.009
Years of menopause	−0.084	0.22
Year(s) of education	0.110	0.11
PSQI	0.119	0.085
Physical activity	0.003	0.97
Weight	−0.036	0.61
Height	**0.136** *	0.049

Note:

* *P* < 0.05;

** *P* < 0.01

**Table 5:** The direct and interaction effect of PRAL and CMS on bone resorption

Variables	Step 1			Step 2			Step 3		

	Beta	*t*	*P*	Beta	*t*	*P*	Beta	*t*	*P*
Age (years old)	−0.145	−2.002	**0.047**	−0.155	−2.154	**0.032**	−0.153	−2.117	**0.036**
Year(s) of education	0.080	1.135	0.258	0.102	1.429	0.155	0.102	1.429	0.155
Height	0.098	1.402	0.162	0.106	1.512	0.132	0.106	1.510	0.133
Sleep quality	0.147	2.162	**0.032**	0.158	2.313	**0.022**	0.157	2.305	**0.022**
Serum of 25(OH) vitamin D	−0.078	−1.148	0.252	−0.089	−1.310	0.192	−0.089	−1.309	0.192
PRAL score				0.142	2.096	**0.037**	0.158	1.794	0.074
CMS (0 = normal, 1 = yes)				0.042	0.606	0.545	0.055	0.652	0.515
PRAL*CMS							−0.028	−0.274	0.784

Notes: Step 1: *F* (6,204) = 3.207, *P* = 0.008, *R*^2^ = 0.073; Step 2: *F* (8,202) = 2.996, *P* = 0.005, Δ*R*^2^ = 0.021, Δ*F* (2,202) = 2.362, *P*_(Δ_*_F_*_)_ = 0.097; Step 3: *F* (9,201) = 2.619, *P* = 0.010, Δ*R*^2^ = 0.000, Δ*F* (1,201) = 0.075, *P*_(Δ_*_F_*_)_ = 0.784
